# Predictors of SOZ localization, subsequent surgical intervention, and seizure outcomes in iEEG

**DOI:** 10.1002/acn3.52084

**Published:** 2024-06-03

**Authors:** Rohan Jha, Melissa M. J. Chua, Rani Sarkis, Steven Tobochnik, John D. Rolston

**Affiliations:** ^1^ Harvard Medical School Boston Massachusetts USA; ^2^ Department of Neurosurgery Brigham and Women's Hospital, Harvard Medical School Boston Massachusetts USA; ^3^ Department of Neurology Brigham and Women's Hospital, Harvard Medical School Boston Massachusetts USA

## Abstract

**Objective:**

A third of the patients who undergo intracranial EEG (iEEG) for seizure‐onset zone (SOZ) localization do not proceed to resective surgery for epilepsy, and over half of those who do continue to have seizures following treatment. To better identify candidates who are more likely to see benefits from undergoing iEEG, we investigated preoperative and iEEG peri‐operative features associated with the localization of a putative SOZ, undergoing subsequent surgical treatment, and seizure outcomes.

**Methods:**

We conducted a retrospective cohort study of consecutive patients who underwent iEEG from 2001 to 2022 at two institutions. Outcomes included SOZ identification, proceeding to surgical treatment (resection vs. neuromodulation), and subsequent seizure freedom.

**Results:**

We identified 329 unique patients who were followed for a median of 3.9 (IQR:7) years, with a minimum of 2‐year follow‐up for seizure outcomes analyses. Multivariate analysis identified lateralized and lobar localization on scalp EEG (OR 3.8, *p* = 0.001) to be associated with SOZ localization. Patients with unilateral localization on scalp EEG (OR 3.0, *p* = 0.003), unilateral preimplantation hypothesis (OR 3.1, *p* = 0.001), and lesional preoperative MRI (OR 2.1, *p* = 0.033) were more likely to undergo resection than neuromodulation. Similarly, a unilateral pre‐implantation hypothesis (OR 2.6, *p* < 0.001) favored seizure freedom, whereas prior neuromodulation (OR 0.3, *p* = 0.013) decreased the odds. Larger number of preoperative anti‐seizure medications (ASMs) did not influence seizure freedom rates but did decrease favorable (Engel I, II) seizure outcomes (OR 0.7, *p* = 0.026).

**Interpretation:**

Non‐invasive localization data prior to iEEG are associated with subsequent resection and seizure freedom, independent of iEEG localization. Factors predictive of SOZ localization are not necessarily predictive of post‐operative seizure freedom.

## Introduction

For patients with medically refractory epilepsy, surgical intervention is an effective and safe option to improve seizure control and quality of life in appropriately selected candidates.^1^, ^2^, ^3^ However, epilepsy surgery remains one of the most underutilized evidence‐based treaments.^4^ Patient beliefs and systemic factors have biased patients against surgery in intractable cases.^5^ This discrepancy is widening further, as the indications for epilepsy surgery have expanded. More complex epilepsy manifestations are now being treated, with an expansion of pathologies and etiologies including those without lesions or radiologic abnormalities.^6^, ^7^, ^8^

Successful surgical intervention is contingent on the accurate identification of the seizure‐onset zone (SOZ), a key predictor of the putative epileptogenic zone (EZ). As a result, there has been increasing utilization of intracranial electroencephalography (iEEG),^8^ driven primarily by stereoelectroencephalography (SEEG) in recent years, to better identify and localize the SOZ in complex epilepsy manifestations. In some patients, a SOZ cannot be well‐delineated.^9^ Up to a quarter of patients undergoing iEEG do not complete resective intervention.^10^, ^11^ Of those who do, around a half do not maintain seizure freedom.^12^ Hence, identifying appropriate candidates for iEEG is nuanced; though epilepsy surgery should be offered to maximize quality of life, undue surgical morbidity should be minimized.

Nomogram models to predict seizure freedom rates following resection have been established, however, these prediction scales are not comprised exclusively of patients requiring iEEG.^13^ Furthermore, with the advent of responsive neurostimulation (RNS) and other neuromodulation techniques, meaningful seizure reduction and brief periods of seizure remission are often achieved, although sustained seizure freedom is unlikely.^14^ Hence, characterizing success after iEEG and epilepsy surgery is a complex consideration. Patient‐specific factors, strength of the EZ hypothesis, implant plan, and surgical strategy all likely influence success in SOZ localization to guide subsequent surgery and optimize seizure outcomes.

Extended follow‐up data have suggested that seizure freedom decreases over time, with only a third of the patients undergoing resective surgery remaining seizure free at 10 years.^15^, ^16^, ^17^ We hypothesize that a well‐delineated SOZ is predictive of subsequent surgical intervention and favorable (Engel I, II) seizure outcomes, but not long‐term sustained seizure freedom. To evaluate these outcomes after iEEG, we analyze a large retrospective cohort of patients who underwent iEEG with SEEG and/or subdural grids and strips (SDE). We identify preoperative and iEEG factors associated with (a) identification of a well‐delineated SOZ, (b) rate of subsequent treatment, including resection and neuromodulation, and (c) seizure freedom and favorable seizure outcomes.

## Methods

### Patient selection

We carried out a multicenter retrospective cohort observational study, including patients with medically refractory epilepsy who underwent evaluation with iEEG from 2001 and 2022 at the Mass General Brigham (MGB) hospital system. Patients underwent iEEG procedures if it was thought to be necessary for improved seizure localization or mapping of eloquent cortex prior to a therapeutic surgical intervention. Both SEEG and SDE modalities were included in analyses. Patients with epidural electrodes and foramen ovale electrodes were excluded. For patients with multiple iEEG interventions within the MGB system, we only included and analyzed the data from the first iEEG intervention. If patients had undergone prior investigations at other institutions, this feature was noted but patients were not discarded from analyses as we did not have complete data from their prior investigation. The study was approved by the MGB Institutional Review Board.

### Data collection and outcomes ascertainment

We collected demographic data and preoperative clinical information, including age at diagnosis of epilepsy, number of prescribed preoperative anti‐seizure medications (ASMs), seizure frequency per month, and seizure semiology from the electronic medical record. Furthermore, we also identified the results of non‐invasive investigations prior to iEEG, including scalp EEG and MRI findings. In addition, we also collected data about iEEG investigation parameters, perioperative complications, subsequent resective or neuromodulatory interventions for treatment, and seizure freedom rates at last follow‐up. Cases with missing data were excluded. Both adult and pediatric patients were included in analyses.

Primary outcomes included likelihood of SOZ identification, likelihood of subsequent neurosurgical intervention, and likelihood of seizure freedom as a function of preoperative and iEEG intervention factors. Successful identification of the SOZ was defined as unambiguous iEEG electroclinical onsets within a specific sublobar area, as documented by multidisciplinary conference consensus, regardless of any subsequent surgical treatment.

Complications that were identified included wound or intracranial infections, subdural hematomas, radiographic intraparenchymal hemorrhage (IPH), and infarctions. There was no mortality following iEEG, so this was not analyzed. Patients with a follow‐up with at least 2 years were included in seizure outcome analyses. We defined seizure freedom as Engel class I outcomes in the latest outpatient follow‐up clinical note, and favorable seizure outcome as Engel I/II.

### Statistical methods

We used MATLAB 2023a (The MathWorks, Inc., Natick, Massachusetts) for all statistical analyses. We calculated descriptive data using number (%) for nominal data, and median (interquartile range (IQR)) for continuous data. Baseline clinical characteristics were compared using two‐sided Student's *t*‐test for continuous variables, and Pearson's Chi‐squared test for categorial variables. For our primary outcomes, we calculated bivariate associations between baseline factors and (1) whether a SOZ was identified, (2) whether patients underwent subsequent neurosurgical treatment, and (3) whether patients achieved seizure freedom at last follow‐up. We estimated odds ratio in a univariable fashion between baseline factors. Subsequently, built a multivariable logistic regression model using stepwise backward Akaike information criteria (AIC). For the multivariable models, we used *α* = 0.05 for significance, and the receiver operating characteristic curve (AUC) was used to evaluate the discrimination power of the final model.

## Results

### Patient characteristics

A total of 329 unique patients (49% females) underwent iEEG and were followed for a median of 3.9 (IQR: 7) years. 176 (53.5%) underwent SEEG, 60 (18.2%) underwent SDE, and 93 (28.3%) underwent hybrid approaches using both SDE and depth electrodes. The median age of onset and duration of epilepsy were 15 (IQR: 18) and 16 (IQR: 18) years, respectively. At the time of surgery, patients were a median of 32 (IQR: 19) years old and were taking a median of 3 (IQR: 1) ASMs. They reported a median of 8 (IQR: 27.5) seizures per month, with 264 (80.2%) reporting a prior history of bilateral tonic–clonic seizures. 54 (16.4%) had undergone prior invasive monitoring, 58 (17.6%) had undergone prior resective intervention, and 46 (14.0%) had undergone prior neuromodulatory intervention, such as vagal nerve stimulation (VNS), and responsive neurostimulation (RNS).

### Non‐invasive evaluation

Prior to iEEG evaluation, patients underwent non‐invasive evaluation with scalp EEG and MRI, with some undergoing additional investigations with PET (74.8%). 219 (66.6%) had a suspected causative lesion by MRI. On scalp EEG, 241 (73.9%) of patients had seizures with clear lateralization and lobar localization, with 95 (39.4%) being right‐sided, 103 (42.7%) being left‐sided, and 43 (17.8%) bilateral.

### iEEG implantation details and peri‐operative safety

The pre‐implantation hypothesis was bilateral in 166 (50.5%), and hence they underwent bilateral iEEG exploration. Recording occurred for a median of 8 (IQR: 3) days, and it took a median of 2 (IQR: 3) days prior to the first detected seizure. iEEG complications were uncommon, with no mortality and a total of 56 (17%) patients with complications. Specifically, 21 (6.4%) had infections, 17 (5.1%) had subdural hematomas, 14 (4.3%) had radiographic IPH, and 11 (3.3%) had infarcts. Of these patients, 18 (5.5%) were taken back to the operating room for management of their complications. Some patients, 8 (2.4%), also required re‐positioning of their electrodes. Patients were in the hospital during their admission for a median of 11 (IQR: 7) days, which often included a resection at the time of the electrode removal in SDE and hybrid patients.

### Identification of putative SOZ

**Figure 1:**
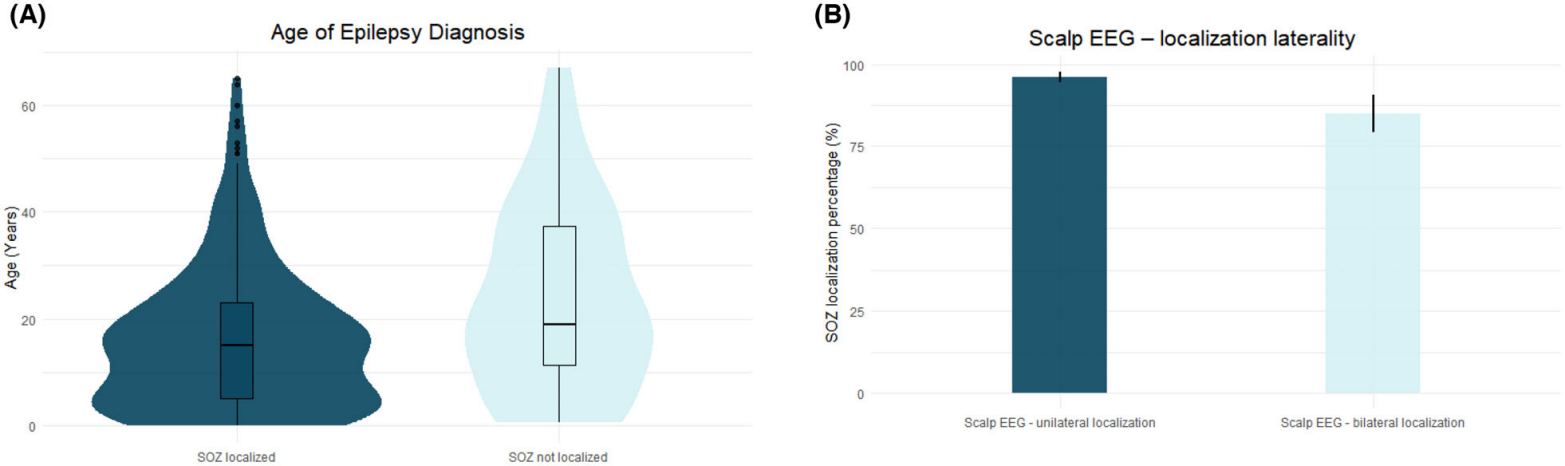
Predictors of iEEG SOZ localization. (A) The age of epilepsy diagnosis and (B) unilateral seizure localization on scalp EEG were both predictive of SOZ identification on iEEG on multivariable analyses.

The presumed SOZ was identified in 293 (89.1%) of patients. In 55 patients (18.8%), multifocal SOZ were identified. Of the patients with unifocal SOZs, there was an even distribution across left (50.0%) and right (50.0%) hemispheres. Preoperative and iEEG peri‐operative variables were assessed for differences between patients for whom the SOZ was identified and not (Table 1). In the univariate analysis, the SOZ was more likely to be identified in patients who were younger at age of epilepsy diagnosis, had a preoperative PET scan carried out and had unilateral lobar localization on scalp EEG. A multivariable logistic model chosen by stepwise AIC showed statistical significance (*p* < 0.0001), with an AUC of 0.70 for all patients who underwent iEEG. Two significant variables were identified, namely seizures with unilateral localization on scalp EEG (odds ratio: 3.8, confidence interval: 1.8, 8.3, *p* = 0.001), and age of epilepsy diagnosis (OR: 0.97, CI: 0.95, 0.95, *p* = 0.021) (Fig. 1).

**Table 1:** Predictors of SOZ localization.

Feature	iEEG (*n* = 329)			
	*p*‐value	OR	SOZ localized	SOZ not localized
*Univariate analyses*				
Age at epilepsy diagnosis (years)	**0.018**	**0.97 (0.95, 0.99)**	15	19
Duration of epilepsy	0.285			
Age at surgery	0.159			
Pre‐op number of ASMs	0.111			
Pre‐op seizure frequency	0.877			
Male sex	0.179			
Presence of GTCs	0.580			
Prior invasive monitoring	0.275			
Prior resective intervention	0.206			
Prior neuromodulation	0.824			
Pre‐op MRI abnormal	0.286			
Pre‐op PET carried out	**0.018**	**2.3 (1.2, 4.6)**	76%	61%
Localization on scalp EEG	**<0.0001**	**3.6 (1.8, 7.2)**	77%	50%
Scalp EEG—unilateral localization	**<0.001**	**4.0 (1.9, 8.5)**	64%	31%
iEEG investigation—unilateral	0.073			
iEEG length of monitoring	0.460			
*Multivariable analyses. AUC = 0.701*				
Age at epilepsy diagnosis (years)	**0.021**	**0.97 (0.95, 0.99)**	15	19
Scalp EEG—unilateral localization	**0.001**	**3.8 (1.8, 8.3)**	64%	31%

All bolded are *p* < 0.05 (highlighted for viewability)

### Subsequent surgery for treatment

We next asked what features in patients undergoing iEEG could predict the type of subsequent surgery offered. Of the total iEEG cohort, 275 (84.1%) were offered and underwent subsequent neurosurgical intervention for epilepsy treatment. Resection was carried out in 206 (74.6%) of patients, whereas Laser interstitial thermal therapy (LITT) was carried out in 17 patients (6.2%). Responsive neurostimulation (RNS) was carried out in 44 (15.9%) of patients, VNS in 7 (2.5%) and DBS in one (0.4%). Finally, one patient (0.4%) underwent multiple subpial transections (MST).

**Figure 2:**
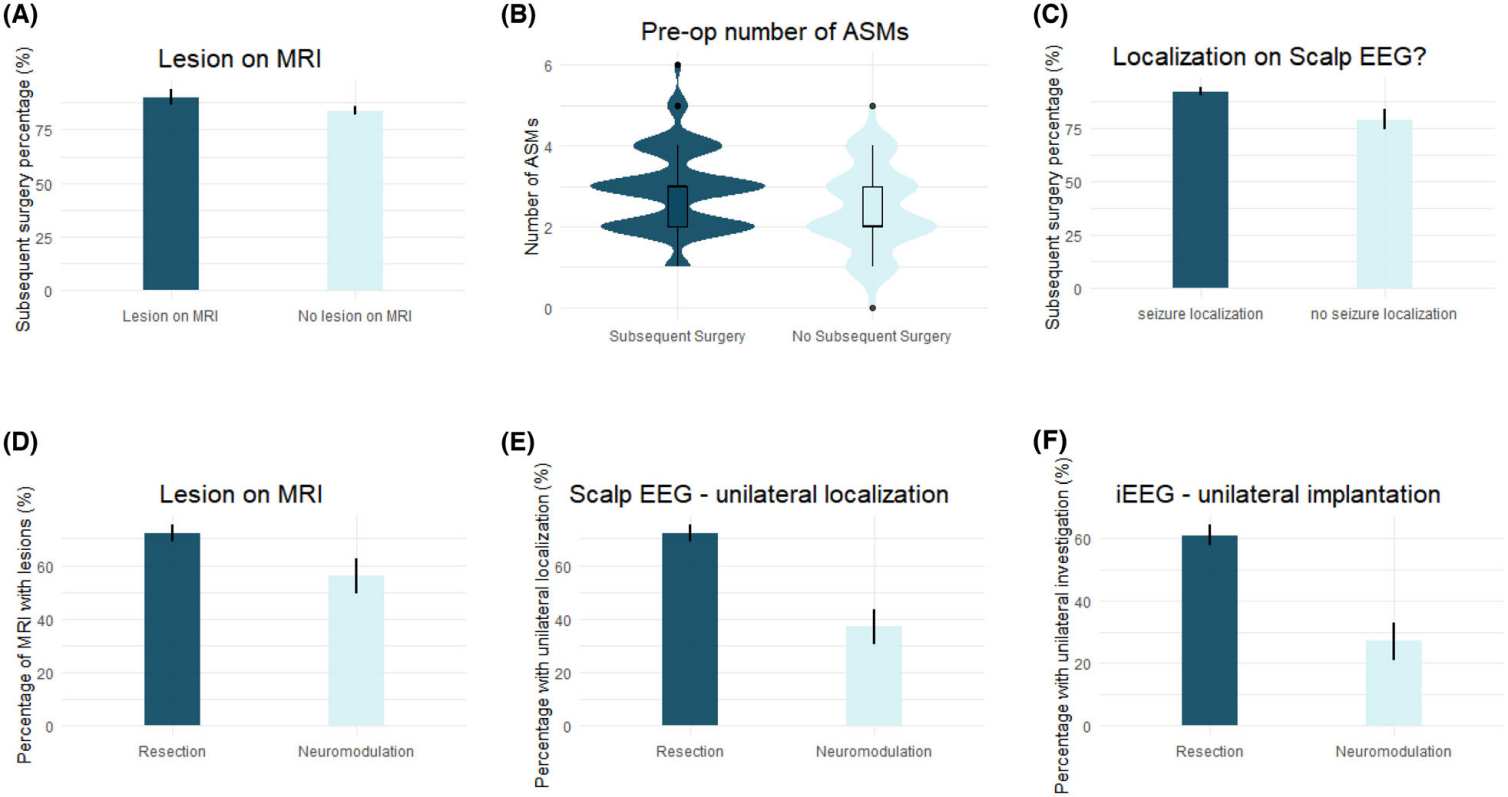
Predictors of subsequent surgical treatment. The (A) presence of a lesion on the preoperative MRI, (B) preoperative number of ASMs, and (C) seizure‐onset localization on scalp EEG all significantly undergoing neurosurgical intervention for treatment after iEEG monitoring on multivariable analyses. The (D) presence of a lesion on the preoperative MRI, (E) having a unilateral seizure onset localization on scalp EEG, and (F) undergoing unilateral iEEG implantation were all predictors of undergoing resection versus neuromodulation on multivariable analyses.

The results of univariable analysis, with respect to likelihood of undergoing subsequent treatment, are shown in Table 2. On multivariable analysis, the best model (*p* < 0.0001, AUC = 0.82) identified increased preoperative number of ASMs (OR: 1.5, CI: 1.1–2.1, *p* = 0.031), abnormality on preoperative MRI (OR: 2.1, CI: 1.1–4.1, *p* = 0.036), and localization on scalp EEG (OR: 2.3, CI: 1.1–4.6, *p* = 0.021) as significant predictors of undergoing neurosurgical intervention for treatment after iEEG monitoring (Fig. 2A–C).

**Table 2:** Predictors of undergoing subsequent surgical treatment.

Feature	iEEG (*n* = 329)			
	*p*‐value	OR	Subsequent surgical treatment	No further surgical treatment
*Univariate analysis*				
Age at epilepsy diagnosis (years)	**0.002**	**0.97 (0.95, 0.99)**	14	19
Duration of epilepsy	0.102			
Age at surgery	0.086	0.98 (0.96, 1.00)		
Pre‐op number of ASMs (*n*)	**0.005**	**1.6 (1.1, 2.2)**	3	2
Pre‐op seizure frequency	0.253			
Male sex	0.059	0.6 (0.3, 1.0)		
Presence of GTCs	0.255			
Prior invasive monitoring	0.519			
Prior resective intervention	0.227			
Prior neuromodulation	0.318			
Pre‐op MRI abnormal	**0.039**	**1.9 (1.1, 3.4)**	69%	54%
Pre‐op PET carried out	0.738			
Localization on scalp EEG	**0.002**	**2.6 (1.4, 4.9)**	72%	42%
Scalp EEG—unilateral localization	**<0.0001**	**4.3 (2.3, 8.2)**	66%	31%
iEEG investigation—unilateral	**0.009**	2.3 (1.2, 4.3)		
iEEG length of monitoring	**0.040**	**0.94 (0.90, 0 99)**	8	9
Days before seizure onset	0.079	0.91 (0.82, 1.01)		
Complications	0.191			
*Multivariable analyses. AUC = 0.82*				
Pre‐op number of ASMs (*n*)	**0.031**	**1.5 (1.1, 2.1)**	3	2
Pre‐op MRI abnormal	**0.036**	**2.1 (1.1, 4.1)**	69%	54%
Localization on scalp EEG	**0.021**	**2.3 (1.1, 4.6)**	77%	56%

When comparing patients who underwent subsequent resection as compared to neuromodulation, multiple significant predictors were identified on univariable analysis (Table 3). On multivariable analysis, the best model (*p* < 0.0001, AUC: 0.76) identified abnormality on preoperative MRI (OR: 2.1, CI: 1.1–4.1, *p* = 0.033), unilateral localization on scalp EEG (OR: 3.1, CI: 1.6–6.2, *p* = 0.001), and unilateral iEEG investigation (OR: 3.0, CI: 1.5–6.3, *p* = 0.003) as predictors favoring resection over neuromodulation (Fig. 2D–F).

**Table 3:** Predictors of undergoing resection versus neuromodulation.

Feature	iEEG (*n* = 258)			
	*p*‐value	OR	Resection	Neuromodulation
*Univariate analyses*				
Age at epilepsy diagnosis	0.117			
Duration of epilepsy	0.437			
Age at surgery	0.178			
Pre‐op number of ASMs	0.860			
Pre‐op seizure frequency	0.350			
Male sex	0.155			
Presence of GTCs	0.061			
Prior invasive monitoring	0.641			
Prior resective intervention	0.832			
Prior neuromodulation	0.408			
Pre‐op MRI abnormal	**0.023**	**2.1 (1.1, 3.9)**	72%	56%
Pre‐op PET carried out	0.377			
Localization on scalp EEG	**0.017**	**2.2 (1.2, 4.3)**	80%	63%
Scalp EEG—unilateral	**<0.0001**	**4.4 (2.4, 8.4)**	72%	37%
iEEG investigation—unilateral	**<0.0001**	**4.2 (2.1, 8.2)**	61%	27%
iEEG length of monitoring	0.942			
Days before seizure onset	0.281			
Complications	0.718			
*Multivariable analyses. AUC = 0.76*				
Pre‐op MRI abnormal	**0.033**	**2.1 (1.1, 4.1)**	72%	56%
Scalp EEG—unilateral	**0.001**	**3.1 (1.6, 6.2)**	72%	37%
iEEG investigation—unilateral	**0.003**	**3.0 (1.5, 6.3)**	61%	27%

### Seizure outcomes

**Figure 3:**
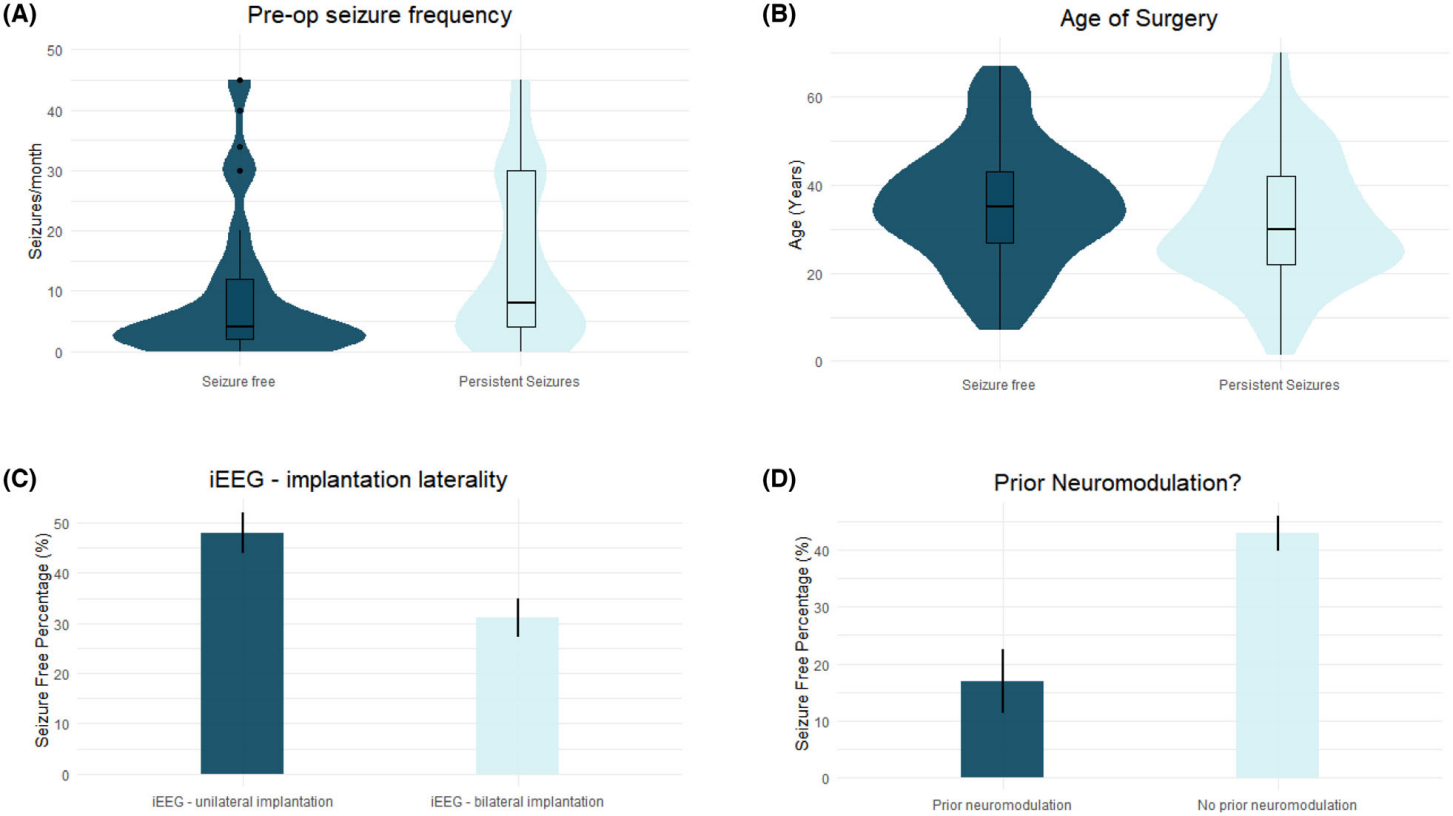
Predictors of seizure freedom. The (A) preoperative seizure frequency per month, (B) age at surgery, (C) undergoing unilateral iEEG implantation, and (D) having undergone prior neuromodulation all significantly predicted favorable seizure freedom on multivariable analysis.

Preoperative and peri‐operative variables were assessed for differences between those who achieved Engel I outcomes, and those who did not. Overall, 126 (40.1%) had Engel Class I outcome after a median of 3.9 (IQR: 7, range: 2, 22) years follow‐up. Significant variables on univariate analysis across iEEG cohort and sub‐cohorts are shown in Table 4. On multivariable analysis (*p* < 0.0001, AUC: 0.71), older age at surgery (OR: 1.02, 1.0–1.04), preoperative seizure frequency (OR: 0.99, CI: 0.98–0.99, *p* = 0.026), prior neuromodulation (OR: 0.3, CI: 0.1–0.8, *p* = 0.013), and unilateral iEEG investigation (OR: 2.6, CI: 1.5–4.5, *p* = 0.008) were significantly associated with seizure freedom (Fig. 3).

**Table 4:** Predictors of seizure freedom.

Feature	iEEG (*n* = 284)			
	*p*‐value	OR	Seizure freedom	Seizure persistence
*Univariate analyses*				
Age at epilepsy diagnosis (years)	**0.005**	**1.02 (1.01,104)**	18	13
Duration of epilepsy	0.285			
Age at surgery (years)	**0.030**	**1.02 (1.0, 1.04)**	35	30
Pre‐op number of ASMs	**0.004**	**0.7 (0.6, 0.9)**	3	3
Pre‐op seizure frequency (seizures per month)	**0.020**	**0.99 (0.99, 1.00)**	4	11
Male sex	0.081	1.5 (0.96, 2.36)		
Presence of GTCs	0.540			
Prior invasive monitoring	0.486			
Prior resective intervention	0.874			
Prior neuromodulation	**0.002**	**0.3 (0.1, 0.6)**	6%	20%
Pre‐op MRI abnormal	0.271			
Pre‐op PET carried out	0.896			
Localization on scalp EEG	0.769			
Scalp EEG—unilateral	0.282			
iEEG investigation—unilateral	**<0.001**	**2.5 (1.5, 3.9)**	63%	42%
iEEG length of monitoring	0.614			
Days before seizure onset	0.664			
Complications	0.316			
*Multivariable analyses. AUC = 0.71*				
Age at surgery (years)	**0.041**	**1.02 (1.0, 1.04)**	35	30
Pre‐op seizure frequency (seizures per month)	**0.026**	**0.99 (0.98, 0.99)**	4	11
Prior neuromodulation	**0.013**	**0.3 (0.1, 0.8)**	6%	20%
iEEG investigation—unilateral	**<0.001**	**2.6 (1.5, 4.5)**	63%	42%

**Figure 4:**
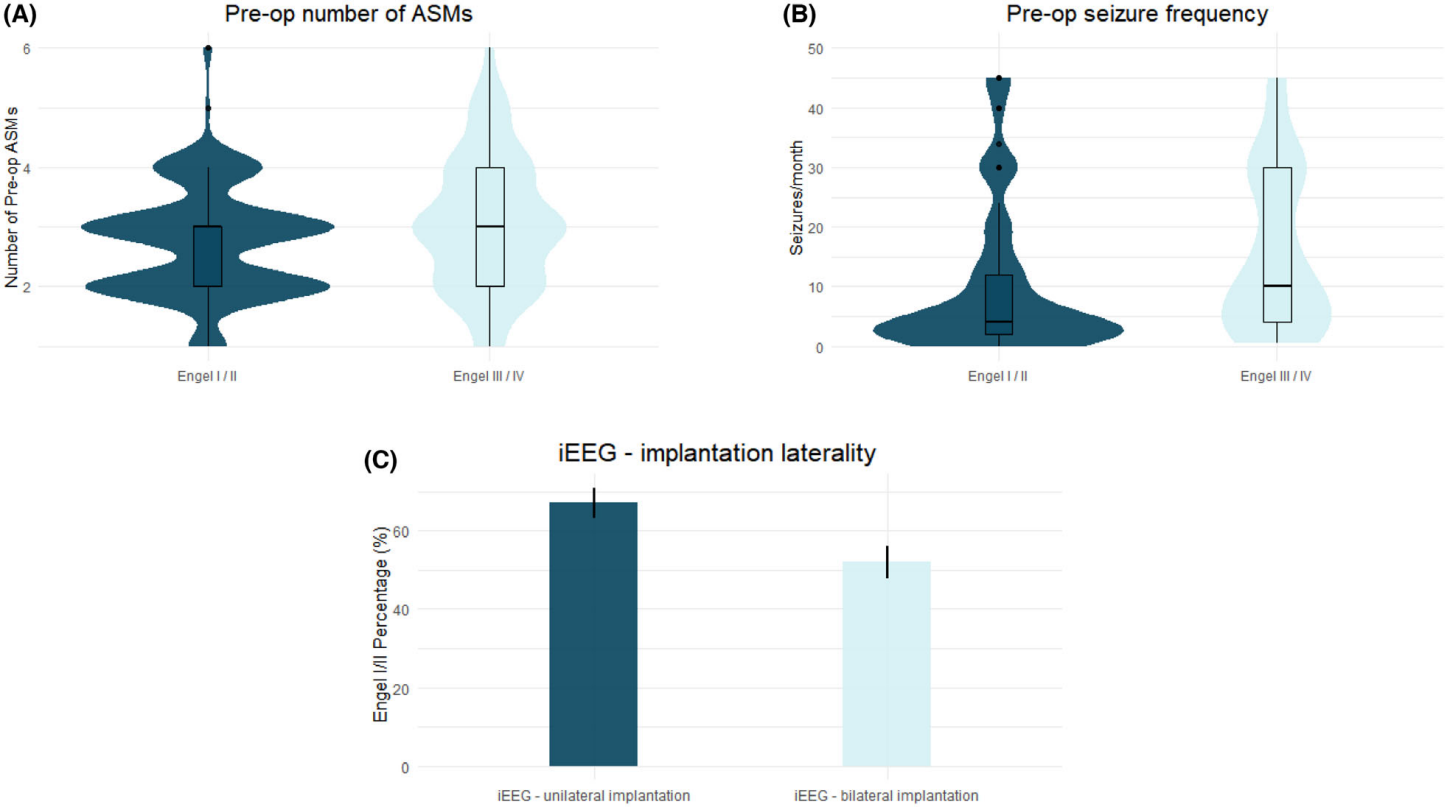
Predictors of favorable seizure (Engel I/II) outcomes. The (A) preoperative number of ASMs, (B) preoperative seizure frequency per month, and (C) undergoing unilateral iEEG implantation all significantly predicted favorable seizure outcomes on multivariable analysis.

When evaluating predictive factors with respect to favorable (Engel I/II) seizure outcomes, three factors remained significant on multivariable analyses (*p* < 0.001, AUC: 0.68): preoperative number of ASMs (OR: 0.7, CI: 0.5–0.9, *p* = 0.026), preoperative seizure frequency (OR: 0.99, CI: 0.98–0.99, *p* = 0.009), and unilateral iEEG investigation (OR: 2.2, CI: 1.2–4.1) (Fig. 4, Table 5).

**Table 5:** Predictors of favorable seizure outcomes.

Feature	iEEG (*n* = 284)			
	*p*‐value	OR	Engel I/II	Engel III/IV
*Univariate analyses*				
Age at epilepsy diagnosis (years)	**0.001**	**1.03 (1.01, 1.05)**	16	11
Duration of epilepsy	0.087	1.0 (0.99, 1.02)		
Age at surgery (years)	**0.002**	**1.03 (1.01, 1.05)**	35	29
Pre‐op number of ASMs	**0.001**	**0.7 (0.5, 0.9)**	3	3
Pre‐op seizure frequency	**0.002**	**0.99 (0.98, 0.99)**	4	20
Male sex	0.174			
Presence of GTCs	0.425			
Prior invasive monitoring	**0.040**	**0.5 (0.3, 0.97)**	13%	24%
Prior resective intervention	0.282			
Prior neuromodulation	**<0.001**	**0.3 (0.2, 0.6)**	8%	24%
Pre‐op MRI abnormal	0.890			
Pre‐op PET carried out	0.599			
Localization on Scalp EEG	**0.017**	**1.9 (1.1, 3.1)**	83%	68%
Scalp EEG—unilateral localization	**0.019**	**1.7 (1.1, 2.8)**	88%	81%
iEEG investigation—unilateral	**0.015**	**1.8 (1.1, 2.8)**	57%	42%
iEEG length of monitoring	0.760			
Days before seizure onset	0.833			
Complications	0.598			
*Multivariable analyses. AUC = 0.68*				
Pre‐op number of ASMs	**0.026**	**0.7 (0.5, 0.9)**	3	3
Pre‐op seizure frequency	**0.009**	**0.99 (0.98, 0.99)**	4	20
iEEG investigation—unilateral	**0.015**	**2.2 (1.2, 4.1)**	57%	42%

## Discussion

Determining prognostic factors is critical in guiding the complex decision‐making process of selecting appropriate candidates to undergo iEEG for epilepsy surgery. As highlighted earlier, up to a quarter of patients undergoing SEEG do not progress to resective intervention, and of those who do, only about a half maintain seizure freedom. Recognizing the suboptimal outcomes in a significant portion of the patients undergoing iEEG and subsequent treatment highlights the need for elucidating the underlying drivers of these outcomes. Ideally, advancements in research, innovation, clinical practice, and surgical technique would use the knowledge of these drivers to improve outcomes. In the interim, improving patient selection can inform resource allocation and enhance patient counseling and management.

Here, we report on a large retrospective series of 329 patients with drug‐resistant epilepsy who underwent iEEG monitoring, and identified preoperative and iEEG variables that are predictive of SOZ localization, proceeding to further therapeutic surgery, and subsequent seizure freedom. Although a few prior reports have described these associations, these studies have primarily evaluated only included resection cases, and/or only reported on seizure freedom.^12^, ^18^, ^19^, ^20^, ^21^ In this study, we report on outcomes with respect to neuromodulation and meaningful seizure reduction, which has not been done previously.

### SOZ localization

Our findings suggest that lateralized and lobar localization on scalp EEG and younger age at epilepsy diagnosis were independent predictors of SOZ localization, though the influence of the age was modest. The etiologies and semiologies of epilepsy have been known to vary through age,^22^, ^23^ which may underlie part of the observed differences in likelihood. Our second independent predictor is likely more clinically important: bilateral localization on scalp EEG suggests bilateral/multifocal seizure foci or rapid diffuse propagation, which may inherently be harder to detect even via invasive means. Similarly, in a previous study, a preimplantation bilateral seizure‐onset hypothesis has been associated with worse SOZ identification.^20^

Conflicting reports exist on the role of a lesion on preoperative MRI in influencing SOZ localization.^19^, ^20^ Patients who have a clear causative and concordant lesion on MRI often undergo surgery without iEEG. The remaining patients, who have discrepancies between MRI findings and other preoperative investigation results, likely require iEEG prior to surgery. Our study suggests that the presence of a lesion offers no additional significant localizing value, as those with relevant, concordant signal with their MRI findings were already likely screened out of undergoing iEEG.

### Subsequent intervention

In this study, patients were more likely to be offered and undergo therapeutic surgery after iEEG if an abnormality was found on the preoperative MRI, if they had a higher preoperative number of ASMs, and if there was a clearly lateralized and localized scalp EEG. Localization on scalp EEG was found to predict both SOZ localization and undergoing subsequent intervention. Similar to our findings, a previous study found that unilateral SOZ was predictive of undergoing resection.^24^

Similar to our findings, a lesional MRI has been reported to be an independent predictor of undergoing resection.^18^ The fact that the presence of a lesion on MRI was not predictive of SOZ localization, but was predictive of undergoing subsequent surgery suggests that structural lesions are targets that bias toward intervention even in cases with poorly localized seizure onsets. It is possible that lesions in this context may be part of an epilepsy network and their removal improve seizure outcomes. Etiologies such as dual pathologies (e.g., stroke and hippocampal sclerosis) which can lead to secondary epileptogenesis, or diffuse focal cortical dysplasias that are difficult to resect may contribute to this discrepancy, although further study of this hypothesis is warranted.

### Seizure outcomes

Consistent with prior studies, we found patients were much more likely to have seizure freedom following resection than neuromodulation.^3^, ^10^, ^18^, ^20^, ^25^ Lateralization and lobar localization on scalp EEG suggesting a lateralized iEEG hypothesis was predictive for both SOZ localization and seizure freedom. However, having undergone previous neuromodulation decreased the chance for seizure freedom and higher number of preoperative ASMs decreased the chance for favorable seizure outcomes, though these features did not influence SOZ localization.

Completeness of resection has historically been identified as a predictor of seizure outcome,^26^ although some data suggest otherwise.^27^ Patients who fail initial epilepsy surgery have been previously identified as having poorer prognosis in achieving seizure freedom.^19^, ^21^, ^28^ Here, prior neuromodulation proved to be a stronger predictor of poor outcome than having undergone previous resection. Patients who are not candidates for surgical resection, whether it be due to unidentified or multifocal seizure foci or due to involvement of eloquent cortex, are often offered neuromodulation as an alternative. Alternatively, patients who fail resection are also potential candidates for neuromodulation. In both cases, the epileptogenic substrate is likely one of relative “surgical refractoriness.”^28^ Thus, further iEEG and resection in such cases should be done with appropriate counseling of expected long‐term outcomes and preferably when a specific residual EZ is hypothesized.

### Pertinent negative predictors

iEEG length of monitoring was not a significant predictor of any evaluated outcomes. Though one might expect that longer required monitoring may suggest more complex epilepsy manifestations, which are associated with worse outcomes, the lack of association suggests that any signal that length of monitoring may provide with respect to predicting outcomes is likely being driven to a larger degree by other correlated covariates. Similarly, any signal that PET may provide is likely being captured in the relevant scalp EEG findings and MRI findings.

### Optimal candidates

Reliable knowledge of predictive factors for SOZ localization and seizure outcomes can be helpful in identification of patients who will benefit from iEEG intervention. Given the complexity in selecting appropriate candidates for iEEG, we sought to identify whether iEEG and subsequent intervention outcomes were already implicit in the preoperative and operative factors. Though we found multiple predictors, our multivariable models had AUC of ~0.7 to 0.8. These values likely limit the efficacy of nomograms and standardized prediction scales at the individual level. Nevertheless, our findings provide a good framework for patient counseling regarding long‐term seizure outcome expectations after iEEG. Furthermore, we reinforce that non‐invasive localization data prior to iEEG are associated with subsequent resection and seizure freedom, independent of iEEG localization. Thus, it is important that surgical decision‐making weigh both non‐invasive and invasive data to predict the EZ and guide surgical intervention. It is likely that technical advances and improved understanding of epilepsy networks amenable to surgical intervention will both increase treatment options and optimize seizure outcomes in the future.

### Limitations

As this was a retrospective cohort study, we were unable to collect standardized prognostic factors or outcomes in a standardized fashion, relying on medical record review. Though we use multivariable analyses to limit confounding bias, it is impossible to eliminate all sources of selection and institution bias. It remains possible there could be a myriad of other features that we failed to identify that could influence localization, treatment, and seizure outcomes. There has been a change in the epilepsy surgery landscape, where SEEG is being used increasingly in favor of SDE. Our cohort has followed these trends, and thus this transition in practice could have affected outcomes. Though the results presented herein are the reflection of two, high‐volume epilepsy centers with national and international referrals over the course of 2 decades, differences in patient selection processes for iEEG at other institution may limit the generalizability of these findings.

## Author Contributions

*Conceptualization; methodology; formal analysis; data curation; writing—original draft; writing—review and editing; visualization*: Rohan Jha. *Conceptualization; writing—review and editing*: Melissa M. J. Chua, Rani Sarkis, and Steven Tobochnik. *Conceptualization; writing—review and editing; supervision*: John D. Rolston.

## Conflict of Interest Statement

All authors have no other relevant disclosures or conflicts of interest.

## Funding Information

No financial support or funding was provided for the preparation of this manuscript.
